# Bioactive polysaccharides from lotus as potent food supplements: a review of their preparation, structures, biological features and application prospects

**DOI:** 10.3389/fnut.2023.1171004

**Published:** 2023-06-28

**Authors:** Guona Dai, Jiale Wang, Jiamei Zheng, Conglong Xia, Yaping Wang, Baozhong Duan

**Affiliations:** ^1^College of Pharmaceutical Science, Dali University, Dali, China; ^2^Formula-Pattern Research Center, School of Traditional Chinese Medicine, Jinan University, Guangzhou, Guangdong, China

**Keywords:** lotus, polysaccharides, separation, structural features, biological activities, applications, SAR

## Abstract

Lotus is a famous plant of the food and medicine continuum for millennia, which possesses unique nutritional and medicinal values. Polysaccharides are the main bioactive component of lotus and have been widely used as health nutritional supplements and therapeutic agents. However, the industrial production and application of lotus polysaccharides (LPs) are hindered by the lack of a deeper understanding of the structure–activity relationship (SAR), structural modification, applications, and safety of LPs. This review comprehensively comments on the extraction and purification methods and structural characteristics of LPs. The SARs, bioactivities, and mechanisms involved are further evaluated. The potential application and safety issues of LPs are discussed. This review provides valuable updated information and inspires deeper insights for the large scale development and application of LPs.

## 1. Introduction

**Figure 1:**
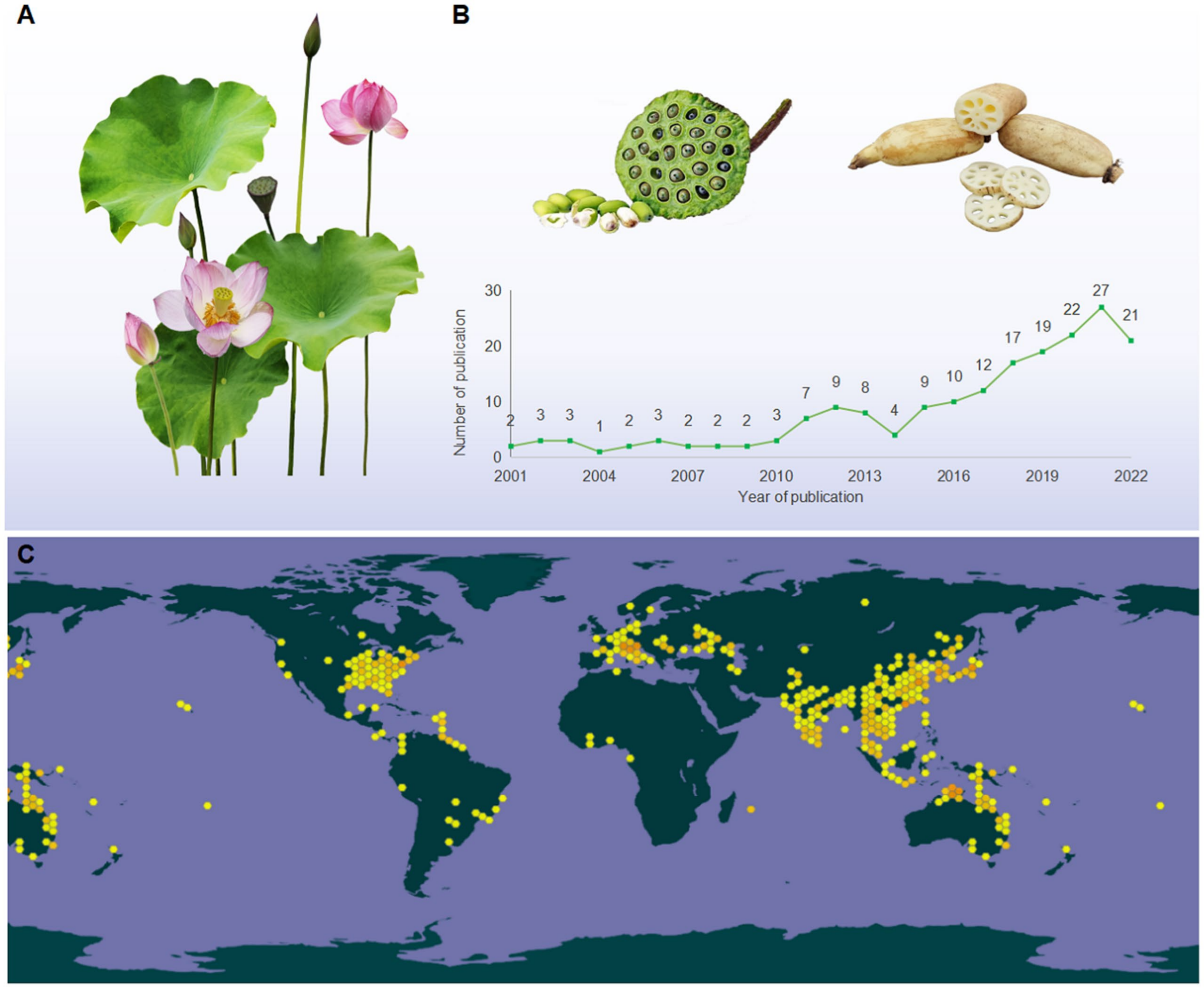
**(A)** The morphological characteristics of lotus; **(B)** the tendency in the number of publications relevant to LPs (2001 from 2022), taken from the database of the web of science (http://apps.webofknowledge.com), topics entered: “lotus polysaccharides” and “*Nelumbo nucifera* polysaccharides”; **(C)** the global distribution map of lotus (https://www.gbif.org/species/2520108).

Lotus, also known as Kamala, water lily, and sacred lotus, is a well-known traditional edible and medicinal aquatic monocotyledonous plant (1), which is broadly distributed in Asia, Oceania, and America (Figure 1C). Its edible parts mainly include seed, root, and leaf (Figure 1A). Owing to its nutritional characteristics, lotus has been used in food for 7,000 years in Asia (2). Since ancient times, various parts of the lotus have been utilized as dietary supplements and herbal medicine in China (3, 4). The medicinal values of lotus can be traced back to the ‘Shen Nong Ben Cao Jing (Shen Nong’s Classic of the Materia Medica),’ written between AD 200–300, which stated that “long-lasting intake of the lotus may lead to agility and longevity” (5). Numerous studies have demonstrated that lotus can be used for many symptoms, such as hypertension, depression, insomnia, cancer, cardiac ailments, and diarrhea (6).

The bioactivities of lotus are closely associated with their phytochemical compositions, which mainly referring to polysaccharides, alkaloids, saponins, and phenolic compounds (1, 6). Among these, polysaccharides have been considered as one of the major bioactive constituents of lotus (6). Previous studies have shown that lotus polysaccharides (LPs) was typical acidic heteropolysaccharide composed mainly of Fuc, Glc, Ara, Gal, Man, Xyl Rha, and GalA with small amounts of Rib, Fru, and GlcA (7). Within the past two decades, various potential bioactivities of LPs have been widely evaluated *in vitro* and *in vivo*, such as antioxidant, immunomodulatory, anti-inflammatory, antidiabetes, antitumor, antibacterial, antiobesity, and antiosteoporosis (8–10). The health benefits associated with LPs, there is a growing interest in their applications in industries focused on functional foods, dietary supplements, pharmaceuticals, and medical products (1).

According to the Web of Science database, a total of 188 articles on LPs have been published during 2001 and 2022 (Figure 1B). These studies primarily focused on extraction, purification, structural characterization, and bioactivity (9, 11, 12). To our knowledge, the most recent review articles regarding the extraction, purification, structures, and bioactivities of LPs have been summarized (7, 13). However, the detailed information on safety issues, structural modification, and structure–activity relationships (SARs) of LPs remain unclear. Moreover, the molecular mechanisms underlying the bioactivities of LPs have not been elaborated, particularly their anti-inflammatory mechanisms and immune regulation. These shortages may hinder the further utilization of LPs. Herein, an overview of the extraction, purification methods, and structural characteristics of LPs are conceived. Besides, the safety issues and structural modification that have never been emphasized are highlighted. Finally, the impact of chemical modification on the biological activity of LPs was comparatively discoursed, and the molecular mechanisms of bioactivities were summarized and discussed.

## 2. Extraction

Extraction is the initial and critical step in preparing active polysaccharides (14). Typically, the extraction procedure of crude LPs is as follows. Firstly, the dried plant materials are crushed into powder and sieved, then colored substances, lipids, and small molecules of the powder are removed by refluxing extraction with petroleum ether or ethanol. Then residues are extracted using water or organic solvents at different temperatures, followed by filtrating. Finally, the filtrates are collected, concentrated, deproteinized, decolorized, dialyzed, and lyophilized.

Conventional methods, including hot water extraction (HWE) (15–20), acidic extraction (21), and alkali extraction (22), have been applied to extract LPs. HWE is the most commonly utilized technique due to its simplicity, safety, and ease of implementation (23). However, this method has several drawbacks, such as low extraction efficiency, significant energy consumption, and extensive time requirements, etc. (24). In contrast, acidic/alkali extraction can enhance extraction efficiency by destroying the structure of plant cell walls structures (23). Nevertheless, adding a dilute acidic or alkali solution can easily cause degradation and reduction of polysaccharides activities (25). In short, although the traditional extraction methods have definite advantages, they also have significant disadvantages.

Novel and effective extraction techniques have been developed to overcome the limitations of these methods. Specifically, ultrasonic-assisted extraction (UAE) (26–28), microwave-assisted extraction (MAE) (29), ultrasound-MAE (UMAE) (30), dynamic high-pressure micro fluidization-assisted extraction (DHPMAE) (31, 32), enzyme-assisted extraction (EAE) (33–35), and deep eutectic solvent-assisted extraction (DESAE) (12, 36) have been implemented to extraction LPs. UAE utilizes the cavitation effect and strong shear forces by applying ultrasound to enhance the extraction ability of LPs (37). This technique leads to shorter extraction time, lower extraction temperatures, and reduced environmental contamination (38). However, applying high-energy ultrasound during extraction can cause permanently alter the structures and activities of LPs due to rapidly forming and collapsing cavitation bubbles within the liquid medium (25).

MAE utilizes microwaves to penetrate plant cells, increase intracellular temperature, and disrupt cell wall structures, thereby improving extraction efficiency. This method offers the advantages of efficiency, energy-conserving, and eco-friendly (39). However, MAE requires expensive equipment and may be unsuitable for large-scale LPs extraction (40). DHPMAE employs collective forces such as shear, high-frequency vibration, cavitation, instantaneous pressure drops, and high pressure (up to 200 MPa) to enhance extraction yield and efficiency (41), which provides mild extraction conditions, a high extraction rate, and reduced impurities. However, it consumes a significant amount of energy (42).

EAE has gained considerable popularity in LPs extraction due to its efficiency, energy-conserving features, and eco-friendly nature. The addition of enzymes promotes the degradation of cell walls and facilitates the release of LPs, resulting in excellent extraction efficiency (43). However, strict control of extraction conditions, including temperature, pH, extraction time, and enzyme dosage, is necessary to implement this method (25). In recent years, DESAE has attracted attention for its low economic cost, safety, biodegradability, and high solvent dissolution ability, which enhances extraction rates by increasing the solubility of LPs in solvents (36). However, it is challenging to separate LPs from solvents, limiting the industrial applications of DESAE (44).

In addition to the extraction method, conditions, such as solid–liquid ratio, extraction temperatures, and extraction times, also influence the extraction rate of LPs (45). Single-factor and orthogonal experiments have indicated that the order of extraction rate is extraction temperature > solid–liquid ratio > extraction time (21). Notably, there are distinct differences in the extraction methods and conditions for different plant parts. HWE has an extraction volume that follows the order: lotus leaf > lotus root > lotus seed > lotus plumula (46, 47). Moreover, the acid/alkali extraction method is exclusively applied to extract LPs from seeds, leaves, and roots, which may be attributed to the presence of acidic or alkaline groups in these polysaccharides (21, 22).

In addition, the yield and bioactivity of LPs are intimately related to their raw materials, extraction methods, and extraction conditions (45). For example, Peng et al. (30) extracted three seed LPs using different methods (HWE, UAE, and UMAE). The results revealed that UMAE exhibited the highest extraction yield (9.78%), followed by HWE (8.13%) and UAE (1.68%), indicating that the choice of extraction method could influence the yield of LPs. Moreover, the extraction methods and conditions can also influence the bioactivities of polysaccharides. Xing et al. (48) extracted four leaf LPs through water, ultrasonic, enzymatic, and alkaline extraction, and subsequently assessed their antioxidant activity. They found that the polysaccharide obtained by water extraction showed the most potent antioxidant activity. Song et al. (16) used amylase, cellulose, pectinase, and protease to extract four leaf LPs (LLEP-A, LLEP-C, LLEP-P, and LLEP-PR) and found that LLEP-P from pectinase extraction significantly improved the immune responses of macrophages *in vitro*. Thus, the selection of an appropriate extraction approach is crucial for obtaining polysaccharides that align with the specific objectives of the experiment.

## 3. Purification

During the process of LPs extraction, impurities, such as proteins, pigments, and other small molecules, are co-extracted (49). Thus, removing co-existing impurities is required before the separation of LPs. Generally, the proteins in LPs were commonly removed by Sevag, trichloroacetic acid, and enzymatic methods (50–52). And the first two approaches have been commonly used to remove the free protein of LPs. However, these two approaches have several shortcomings, such as inefficiency, complicated operation, and a substantial decrease in the content of LPs. Sevag combined with enzymatic methods can overcome these weaknesses (30, 32, 53). Besides, pigments can oxidize LPs, influencing chromatographic and structural analysis. Thus, removing pigments is a critical step in the process of purification. So far, various methods, such as activated carbon adsorption (50), hydrogen peroxide (H_2_O_2_) (54), and microporous resins (29), have been developed to remove the pigments of LPs. H_2_O_2_ and microporous resins were the most common approaches to remove the pigments of LPs, whereas the activated carbon adsorption method is not routinely applied because of the low efficiency and residual effect.

**Figure 2:**
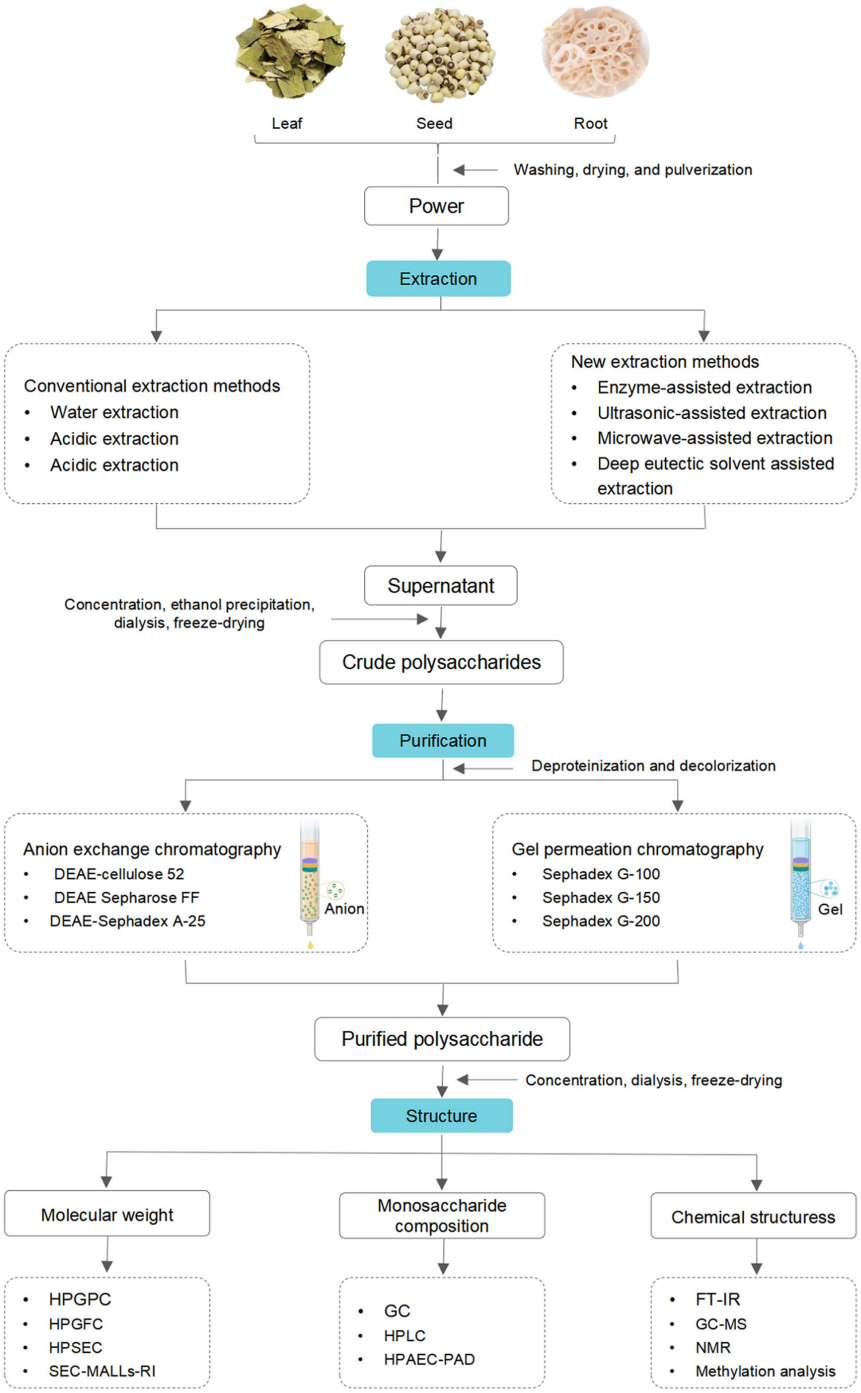
A schematic representation of the extraction, purification, and structure of LPs.

Crude polysaccharides are complex mixtures composed of different degrees of polymerized polysaccharides. Thus, additional purification and separation steps are crucial for investigating their structural characteristics (45). The purification and separation techniques commonly employed for LPs include membrane separation, ethanol precipitation, and column chromatography. In membrane separation, different membranes, such as microfiltration, nanofiltration, ultrafiltration, and classical osmosis membranes, were utilized to separate LPs (55). Ethanol precipitation is the most commonly used method, especially suitable for separating polysaccharides with significant differences in molecular weight (*Mw*) and solubility (56). Column chromatography is one of the most efficient approaches for separating polysaccharides (57). Ion exchange chromatography (IEC) and gel filtration chromatography (GFC) are commonly utilized to purify LPs. Water, different concentrations of sodium chloride (NaCl) solution, or phosphate buffer are frequently employed as eluents in both methods. In general, IEC is employed for purifying neutral or acidic polysaccharides (58), with diethylaminoethyl (DEAE)-cellulose 52, DEAE-Sephadex A-25, and DEAE Sepharose FF being commonly used chromatographic media (9, 52, 59). GFC is commonly used to purify polysaccharides of different *Mw* with Sephadex G-200, Sephadex G-150, or Sephadex G-100 as chromatographic media. Generally, obtaining pure polysaccharides using a single method is challenging. Thus, combining IEC and GFC is often employed to achieve superior purification (40). In summary, the schematic representation of the extraction and purification processes of LPs is shown in Figure 2.

## 4. Structural characteristics

Polysaccharide is one of the vital bioactivity constituents of lotus, which shows diversified and complicated structural characteristics (60). To better understand the LPs, it is necessary to explain their structural characteristics, including *Mw*, monosaccharide composition, and chemical structures. Commonly, LPs’ structural characteristics can be detected by a series of analysis methods, such as high-performance liquid chromatography (HPLC), Fourier-transform infrared spectroscopy (FT-IR), gas chromatography (GC), nuclear magnetic resonance (NMR), methylation analysis, etc. (9, 61, 62). The structural features of LPs from different parts are reviewed, and the relevant information is listed in Table 1.

**Table 1:** Extraction, purification, and structural characterization of LPs from the different parts.

No	Name	Source	Mw (KDa)	Extraction, separation, and purification methods	Monosaccharide compositions	Structural characterization	Analysis techniques	Refs
1	LSSP	Skin of lotus seed	–	90°C distilled water extraction; ethanol precipitation	Rha:Ara:Xyl:Man:Glc:Gal = 2.23:3.47:1.00:3.08:4.27:7.00	–	FT-IR, HPLC, GC	(50)
2	–	Red skin of lotus seed	37.8	50°C and 100°C distilled water extraction; ethanol precipitation; DEAE cellulose and Sephadex G-25 column	Glc:Xyl:Man:Gal	α-pyranose configuration	HPGPC, HPLC, FT-IR, NMR	(51)
3	–	Red skin of lotus seed	49.4	50°C and 100°C distilled water extraction; ethanol precipitation; DEAE cellulose and Sephadex G-25 column	Glc:Xyl:Gal:Fuc:Ara	α-pyranose configuration	HPGPC, HPLC, NMR, FT-IR	(51)
4	SN1	Lotus seed	–	90°C distilled water extraction; ethanol precipitation; Sephadex G-150 column	Ara:Rib:Xyl:Man:Glc:Gal = 1.00:3.60:8.79:7.21:17.28:16.12	Containing α-D-glucopyranosyl ring	GC, FT-IR, AFM	(17, 63)
5	SN2	Lotus seed	–	90°C distilled water extraction; ethanol precipitation; Sephadex G-150 column	Ara:Xyl:Man:Glc:Gal = 3.00:1.28:2.24:12.45:1.00	Backbone of glucan	GC, FT-IR, AFM	(17, 63)
6	SN3	Lotus seed	–	90°C distilled water extraction; ethanol precipitation; Sephadex G-150 column	Fru:Rha:Ara:Rib:Man:Glc:Gal = 6.25:3.45:2.82:1.00:2.34:4.72:14.41	Containing 1 → 3, 1 → 2 or 1 → 4 glycosidic bonds	GC, FT-IR, AFM	(17, 63)
7	–	Lotus seed	4.484	100°C distilled water extraction; ethanol precipitation; DEAE-Sephadex A-25 and Sepharose CL-4B column	D-Rha:D-Xyl:D-Glc:D-Man	α-Glycosidic bond	HPGPC, FT-IR, NMR, HPLC	(55)
8	LPPS	Lotus seed	391	100°C distilled water extraction; ethanol precipitation	Xyl:Glc:Fru:Gal:Fuc	–	HPLC	(64)
9	LSPS-1	Lotus seed	4.484	95°C distilled water extraction; ethanol precipitation; DEAE-Sephadex A-25 and Sepharose CL-4B column	Rha:Ara:Glc:Gal = 7.13:4.81:13.28:1.00	α-pyranose configuration	HPGPC, FT-IR, GC	(59)
10	–	Lotus seed	–	85°C distilled water extraction; ethanol precipitation; DEAE-25 and Sephadex G-10 column	D-Man:D-Glc:D-Gla	Man*p*-(1→), Gal*p*-(1→), α (1 → 6)-Glu*p* and *α* (1 → 6)-Man*p*	GC–MS, HPLC, NMR	(11)
11	LOS3-1, LOS3-2, LOS4	Lotus seed	–	90°C deionized water extraction; ethanol precipitation; hydrophilic interaction liquid chromatography	–	(1 → 6)-α-D-mannopyranosyl and (1 → 6)-α-D-glucopyranosyl with terminal (1 → 2)-α-D-fucopyranosyl and (1 → 6)-α/β-D-glucopyranosyl, the degree of polymerization were 3, 3 and 4	FT-IR, NMR, LC/Q-TOF-MS	(65)
12	ESP-I	Lotus plumula	20.1	82.5°C distilled water extraction; ethanol precipitation; DEAE Cellulose-52 column	D-Xyl:D-Gal:Man = 1.00:7.18:0.51	–	HPGFC, HPLC-ELSD	(52)
13	ESP-II	Lotus plumula	7.94	82.5°C distilled water extraction; ethanol precipitation; DEAE Cellulose-52 column	D-Xyl:D-Ara:D-Gal = 1.00:15.05:8.10:3.05	–	HPGFC, HPLC-ELSD	(52)
14	ESP-III	Lotus plumula	280	82.5°C distilled water extraction; ethanol precipitation; DEAE Cellulose-52 column	–	–	HPGFC, HPLC-ELSD	(52)
15	ELPS-VII	Lotus plumula	–	100°C distilled water extraction;ethanol, precipitation; DEAE Cellulose-32, Sephacryl ™S-200 and Sephadex G-100 column	L-Ara (1.6%), α-D-Xyl (56.5), β-D-Xyl (20.0%), D-Man (5.5%), D-Gal (16.4%)	α and β-pyranose residues, and (1 → 6)-linked glycosyl residue	HPGPC, GC–MS, FT-IR, NMR	(56)
16	LPWF	Lotus plumula	567.6	100°C distilled water extraction; ethanol precipitation; DEAE Sepharose Fast Flow	Rha:Ara:Gal:Xyl:GalA = 7.3:34.0:7.0:19.1:32.6	A pectin linked by RGI and XGA; XGA part: a α-D-1,4-Gal*p*A backbone with t-β-D-Xyl*p* side chains substituted on the O-3 position of Gal*p*A residues; RGI part: (*a* → 4)-α-D-Gal*p*A-(1 → 2)-α-L-Rha*p*-(1 → 4)-α-D-Gal*p*A-(1→) backbone with galactan and arabinan side chains attaching to the Rhap residues	HPLC, GC–MS, NMR, HPLC-QTOF-MS	(9)
17	PNP	Lotus plumula	450	100°C distilled water extraction; ethanol precipitation; DEAE-52 Sepharose Fast Flow and Sephadex G-200 column	Rha (18.41%), GalA (18.24%), Xyl (16.92%), Gal (14.26%), Ara (32.16%)	→5)-α-L-Ara*f*-(1→, →3)-β-D-Gal*p*-(1→, β-D-Xyl*p*-(→1, →3,4)-β-D-Rha*p*-(1→, →4)-β-D-Gal*p*A-(1→	HPGPC, HPLC, FT-IR, NMR	(66)
18	–	Lotus root	17.91	90°C distilled water extraction; ethanol precipitation; HiPrep Sephacryl S-100	Man:Rha:GlcA:GalA:Glc:Gal:Ara = 0.19:0.14:0.08:0.17:6.49:1.00:0.16	[α-D-Glc(1–4)-]_n_	HPLC, SEC-MALLS-RI, FT-IR, NMR	(67)
19	–	Lotus root	–	Distilled water extraction; ethanol precipitation; Sephadex G-100 and G-150 column	Ara:Gal:Man:Glc = 3.0:3.9:1:2.1	–	–	(68)
20	LRP	Lotus root	12.4	90°C distilled water extraction; ethanol precipitation; DEAE-Sepharose FF and Sephadex G-100 column	Glc	α-D-(1 → 4)-linked glucopyranosyl moieties with non-reducing terminal α-D-Glc*p* at O-6 as branches approximately every six residues	HPGPC, GC, GC–MS, FT-IR, NMR, SEM, AFM	(8, 61)
21	LRPs	Lotus root	1.33–5.30	90°C distilled water extraction; ethanol precipitation; HiPrep Sephacryl S-100 gel column	Man:Rha:GalA:Glc:Gal:Ara = 0.19:0.14:0.17:6.49:1.00:0.16	α-(1 → 6)-D-heteroglucans mainly composed of Glc-(1→, →6)-Glc-(1→, →6)-Gal-(1→, →4,6)-Gal-(1 → and →3,6)-Glc-(1 → at a molar ratio of 1.00:4.33:0.83:0.13:1.14	HPSEC-MALLS-RID, HPLC, FT-IR, NMR	(62)
22	LPR1	Lotus root	–	80°C distilled water extraction; ethanol precipitation; DEAE-52 and Sephadex G-25 column	Man (0.12%), Rib (0.18%), GlcA (0.60%), GalA (0.09%), Glc (98.79%), Gal (0.21%)	–	HPLC, FT-IR, NMR, HPSEC-MALLS-RID	(69)
23	–	Lotus root	–	–	Man:Rha:GalA:Glc:Gal:Ara = 0.23:0.09:0.15:16.00:1.00:0.11	–	HPLC-PCD	(70)
24	–	Lotus root peel	–	–	Man:Rib:GalA:Glc:Gal:Ara = 0.09:0.03:0.12:8.88:1.00:0.07	–	HPLC-PCD	(70)
25	–	Lotus root node	–	–	Man:Rib:GalA:GlcA:Gal:Ara = 0.09:0.09:0.05:0.17:3.55:1.00:0.16	–	HPLC-PCD	(70)
26	LB2	Lotus root	18.8	4°C distilled water extraction; ethanol precipitation; Sephadex G-75 column	Man:Rha:Glc:Gal:Xyl = 2:8:7:8:1	–	HPLC, FT-IR, NMR	(71, 72)
27	LRP	Lotus root residue	1.24	90°C distilled water extraction; ethanol precipitation; DEAE-Sepharose Fast Flow and Sephadex G-100 column	Glc	Connecting α-glycosidic bonds	HPGPC, GC–MS, FT-IR, NMR, SEM, AFM	(47)
28	LRP-1, LRP-2	Lotus root residue	–	90°C distilled water extraction; ethanol precipitation; DEAE cellulose-52 and Sephadex G-100 column	Rha:Ara:Glc:Xyl:Gal:Fuc = 5.32:16.03:5.14:1.02:25.98:2.32	–	GC–MS	(73)
29	LP30	Lotus root residue	1.095	90°C distilled water extraction; ethanol precipitation	Glc (91.75%), Gal (8.25%)	–	HPLC, FT-IR, SEC-MALLS-RI	(74)
30	LP45	Lotus root residue	1.416	90°C distilled water extraction; ethanol precipitation	Rha (1.75%), Glc (85.2%), Gal (11.61%), Ara (1.44%)	–	HPLC, FT-IR, SEC-MALLS-RI	(74)
31	LP60	Lotus root residue	1.128	90°C distilled water extraction; ethanol precipitation	Rha (6.39%), Glc (60.08%), Gal (22.76%), Ara (9.98%)	–	HPLC, FT-IR, SEC-MALLS-RI	(74)
32	LP75	Lotus root residue	1.626	90°C distilled water extraction; ethanol precipitation	Rha (5.70%), Glc (81.74%), Gal (12.56%)	–	HPLC, FT-IR, SEC-MALLS-RI	(74)
33	NPh_2_	Lotus root residue	> 2000	90°C distilled water extraction; ethanol precipitation; DEAE-Sepharose Fast Flow and Sepharose CL-6B column	Gal:Ara:Rha:Glc:Fuc:Xyl = 26.74:16.17:5.69:5.49:2.31:1.00	–	HPLC, FT-IR	(75)
34	D-LLP-1	Lotus leaf	549.54	76°C distilled water extraction; DEAE cellulose-52 column	Gal (6.83%), Glc (0.57%), Ara (2.73%), Man (3.25%), Xyl (9.20%), Rha (19.235), Fuc (58.19%)	–	HPGPC, GC, FT-IR	(32)
35	H-LLP-1	Lotus leaf	578.09	76°C distilled water extraction; DEAE cellulose-52 column	Gal (5.03%), Glc(7.04%), Ara (19.39%), Man (2.21%), Xyl (6.07%), Rha (22.82%), Fuc (37.45%)	–	HPGPC, GC, FT-IR	(32)
36	–	Lotus leaf	165.0	65°C distilled water extraction; ethanol precipitation; DEAE cellulose and Sephadex G-200 column	Ara:Man:Glc:Gal = 14:3:3:3	–	GC, HPLC	(54)
37	LLPs-D	Lotus leaf	550	DHPMAE extraction; ethanol and acetone precipitation; DEAE cellulose-52 and Sephadex G-200 column	Rha:Fuc:Ara:Xyl:Man:Glc:Gal = 6.83:0.57:2.73:3.25:9.20:19.23:58.19	–	GC-FID, HPGPC, FT-IR, SEM	(31)
38	LLPs-L	Lotus leaf	578	DHPMAE extraction; ethanol and acetone precipitation; DEAE cellulose-52 and Sephadex G-200 column	Rha:Fuc:Ara:Xyl:Man:Glc:Gal = 5.03:7.04:19.39:2.21:6.07:22.82:37.45	–	GC-FID, HPGPC, FT-IR, SEM	(31)
39	LLWP-1	Lotus leaf	85.1	Distilled water extraction; ethanol precipitation; Sephadex G-100 column	Rha:Ara:Gal:Glu:GalA = 7.0:24.8:28.0:6.0:26.4	–	HPAEC-PAD, HPSEC, FT-IR	(76)
40	LLWP-3	Lotus leaf	12.5	Distilled water extraction; ethanol precipitation; Sephadex G-100 column	Rha:Ara:Gal:Glu:Man:GalA = 6.6:9.8:15.0:8.9:6.2:47.2	–	HPAEC-PAD, HPSEC, FT-IR	(76)
41	NNLP-I-I	Lotus leaf	16.4	HWE; ethanol precipitation; DEAE-Sepharose Fast Flow and Sepharose 6FF column	Ara:Rha:Gal:GalA = 1:1.2:1.2:7.1	A pectic polysaccharide, mainly consisted of a homogalacturonan backbone and rhamnogalacturonan I, containing a long HG region and short RG-I region, with AG-II and 1–3 linked rhamnose as side chains	HPSEC-MALLS, GC–MS, NMR	(77)
42	WNPP-2-RG	Lotus pollen	380	HWE; ethanol precipitation; DEAE-cellulose and Sepharose CL-6B column	Rha (11.5%), GalA (12.0%), Gal (41.2%), Ara (29.75%)	A RG-I type pectin, containing AG-I and AG-II, side chains comprised of 1,5-L-Ara (25.6%), t-D-Gal (12.0%), and 1,6-D-Gal (18.3%)	HPLC, HPGPC, GC–MS, NMR	(78)

### 4.1. Monosaccharide composition

Conventionally, the monosaccharide composition of LPs is determined by HPLC and GC (11, 78). Among newly utilized techniques, high-performance anion-exchange chromatography combined with a pulsed amperometric detector (HPAEC-PAD) is one of the convenient techniques to detect the monosaccharide composition without complex derivatization steps (60). Song et al. (76) used the HPAEC-PAD to determine the monosaccharide composition of two leaf LPs (LLWP-3 and LLWP-1). LLWP-1 consisted of Ara, Glu, Gal, Rha, and GalA (24.8: 6.0: 28.0: 7.0: 26.4), and LLWP-3 consisted of Man, Rha, Ara, Glu, Gal, and GalA (6.2: 6.6: 9.8: 8.9: 15.0: 47.2). As shown in Table 1, LPs mainly consist of Fuc, Glc, Ara, Gal, Man, Xyl, Rha, and GalA (9, 31). Other monosaccharides such as Rib, Fru, and GlcA are also identified in LPs (17, 64, 69). Interestingly, Rib was only detected in the polysaccharides of lotus seed and root (17, 69), while Fru and GlcA were only found in the lotus seed and root, respectively (64, 69). These suggest that LPs exhibited different monosaccharides in different plant parts (i.e., leaf, seed, root, etc.), which leads to differences in polysaccharide properties.

### 4.2. Molecular weight

*Mw* reflects the polymerization of polysaccharides, whose detailed elucidation is crucial to study the physiological properties of polysaccharides (79). Conventionally, size exclusion chromatography (SEC), GFC, and gel permeation chromatography (GPC) were used to determine the *Mw* of LPs (52, 59, 62, 74). Presently, GPC is most frequently used to detect the *Mw* of LPs. Compared to GPC, SEC-MALLs-RI was more efficient and can be applied to measure the polydispersity coefficient, number-average *Mw*, and weight-average *Mw* of polysaccharides (80). For instance, Yan et al. (69) used the SEC-MALLs-RI to determine the *Mw* of the root LPs (LRP-1). The number-average *Mw* and weight-average *Mw* of LRP1 were 251,783 g/mol and 10,236 g/mol, respectively. The LPs were roughly grouped into neutral and acidic fractions, and the *Mw* distribution of LPs was relatively broad, ranging from 1.095 kDa to 578.09 kDa (32, 74). In particular, the *Mw* of root LPs is relatively low. The detailed characteristics are listed in Table 1.

### 4.3. Chemical structure

Currently, the chemical structures of polysaccharides have been detected by FT-IR, GC–MS, and NMR (60). Several studies have isolated LPs with different chemical structures by various separation and purification methods. For instance, Yu et al. (56) obtained homogeneous LPs (ELPS-VII) from the plumula. Its backbone was characterized as α and β-pyranose and (1 → 6)-linked glycosyl residues. Zheng et al. (66) isolated homogeneous LPs (PNP) from plumula. Based on methylation and NMR results, its primary glycosidic linkage was determined as →5)-α-L-Ara*f*-(1→, →3)-β-D-Gal*p*-(1→, β-D-Xyl*p*-(→1, →3,4)-β-D-Rha*p*-(1→, →4)-β-D-Gal*p*A-(1→. Deng et al. (17) separated three LPs (SN1, SN2, and SN3) from seed and found that SN1 consisted of the α-D-glucopyranosyl ring, SN2 mainly consisted of glucan, while SN3 consisted of 1 → 3, 1 → 2, or 1 → 4 glycosidic bonds. Another LPs (LSPS-1) was isolated and purified from seed, whose backbone was mainly composed of α-pyranose configuration (59), which is consistent with Gao et al. (81).

**Figure 3:**
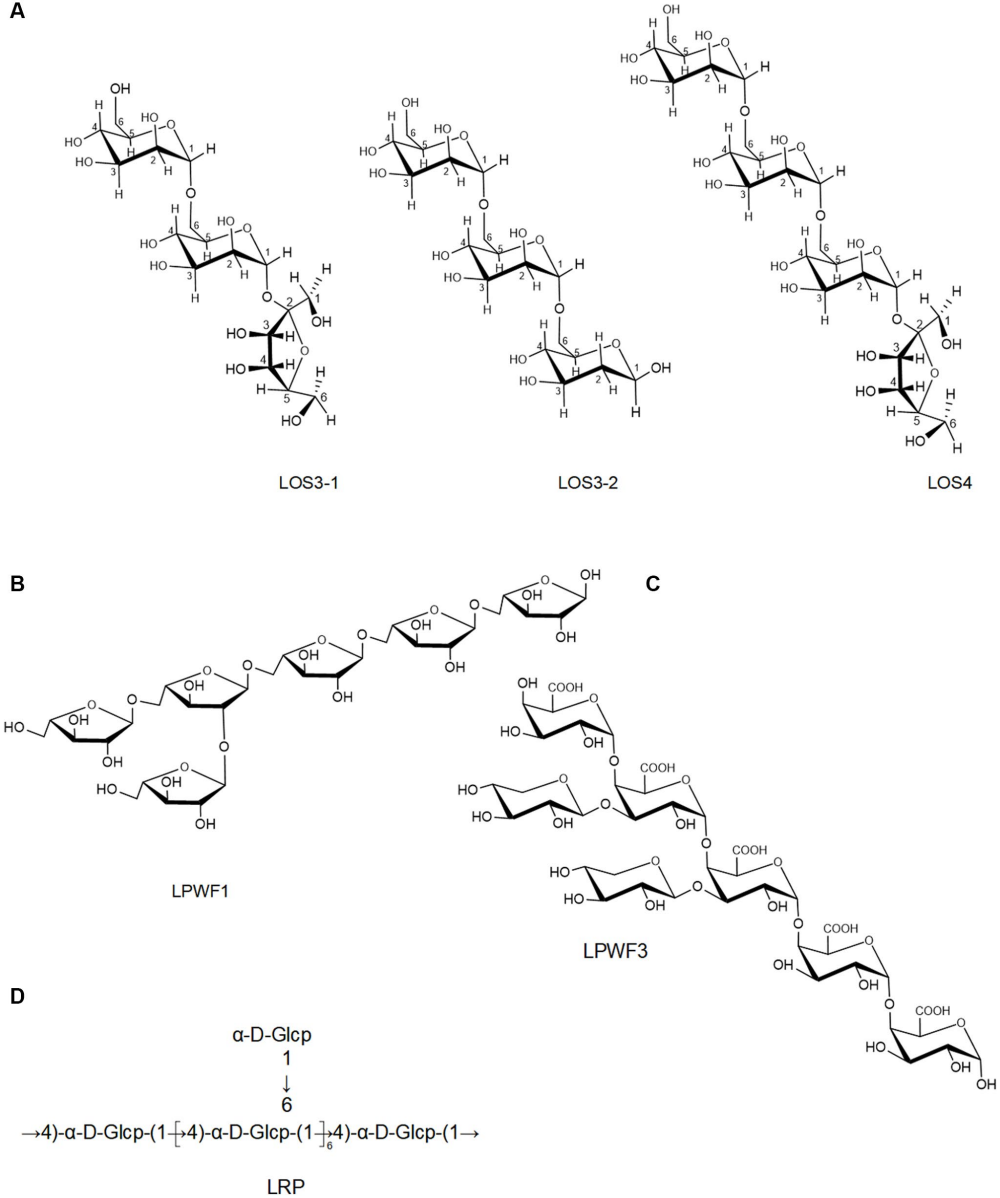
The chemical structures of LPs isolated from the seeds **(A)** and root **(B–D)**.

Moreover, an oligosaccharide from lotus seed was found to contain four glycosidic linkages: α (1 → 6)-Man*p*, α (1 → 6)-Glu*p*, Gal*p*-(1→), and Man*p*-(1→) (11). Lei et al. (65) prepared three oligosaccharides, LOS3-1, LOS3-2, and LOS4, from lotus seed. According to 1D, 2D NMR, and FT-IR, they had a linear structure comprising of (1 → 6)-α-D-glucopyranosyl and (1 → 6)-α-D-mannopyranosyl with terminal (1 → 2)-α-D-fucopyranosyl and (1 → 6)-α/β-D-glucopyranosyl (Figure 3A). The backbone of a root LPs (LRPs) primarily composed of Glc-(1→, →6)-Glc-(1→, →6)-Gal-(1→, →4,6)-Gal-(1 → and →3,6)-Glc-(1 → in the molar ratio of 1.00: 4.33: 0.83: 0.13: 1.14 (62). Another root LPs might be [α-D-Glc (1-4)-]_n_ (67). Another study revealed that root LPs (LRP) was composed of Glc and connected by α-glycosidic bonds (Figure 3D) (47). The root LPs (LRP) obtained by Hu et al. (8) consisted of α-D-(1 → 4)-linked glucopyranosyl moieties with branches attached to O-6 of α-D-Glc*p* residues.

Besides, the structures of pectic polysaccharides from lotus have been elucidated. For instance, Li et al. (78) separated a pectic polysaccharide, WNPP-2-RG, from lotus bee pollen. GC–MS and NMR analysis indicated that WNPP-2-RG comprised a rhamnogalacturonan I (RG-I) region, with the primary linkage consisting of t-D-Gal (12.0%), 1,6-D-Gal (18.3%), and 1,5-L-Ara (25.6%), and side chains were primarily composed of arabinogalactan type I (AG-I) and type II (AG-II). Huang et al. (77) isolated a pectic polysaccharide (NNLP) from lotus leaf, which was predominantly composed of RG-I and homogalacturonan (HG) backbone, including an extended HG fraction and short RG-I fraction with side chains of AG-II and 1–3 linked rhamnose. Recently, She et al. (9) separated a pectic polysaccharide (LPWF) from lotus plumula and further fractionated it into three fractions (LPWF1, LPWF2, and LPWF3) by acid hydrolysis. Methylation and NMR analyses revealed that LPWF represented a unique pectin composition, consisting of xylogalacturonan (XGA, LPWF3) and RGI (RGI, LPWF1-2). LPWF1 was identified as an arabinan peeled from the RGI fraction with a 1,5-linked backbone branching at the O-2 position (Figure 3B). LPWF2 was the residual fraction of RGI consisting of GalA (43.7%), Rha (36.1%), and Gal (17.8%), while LPWF3 was the XGA fraction with a backbone of α-1,4-linked GalA and branches of mono-xylose substitutions attached to O-3 of GalA (Figure 3C).

## 5. Biological activities

**Figure 4:**
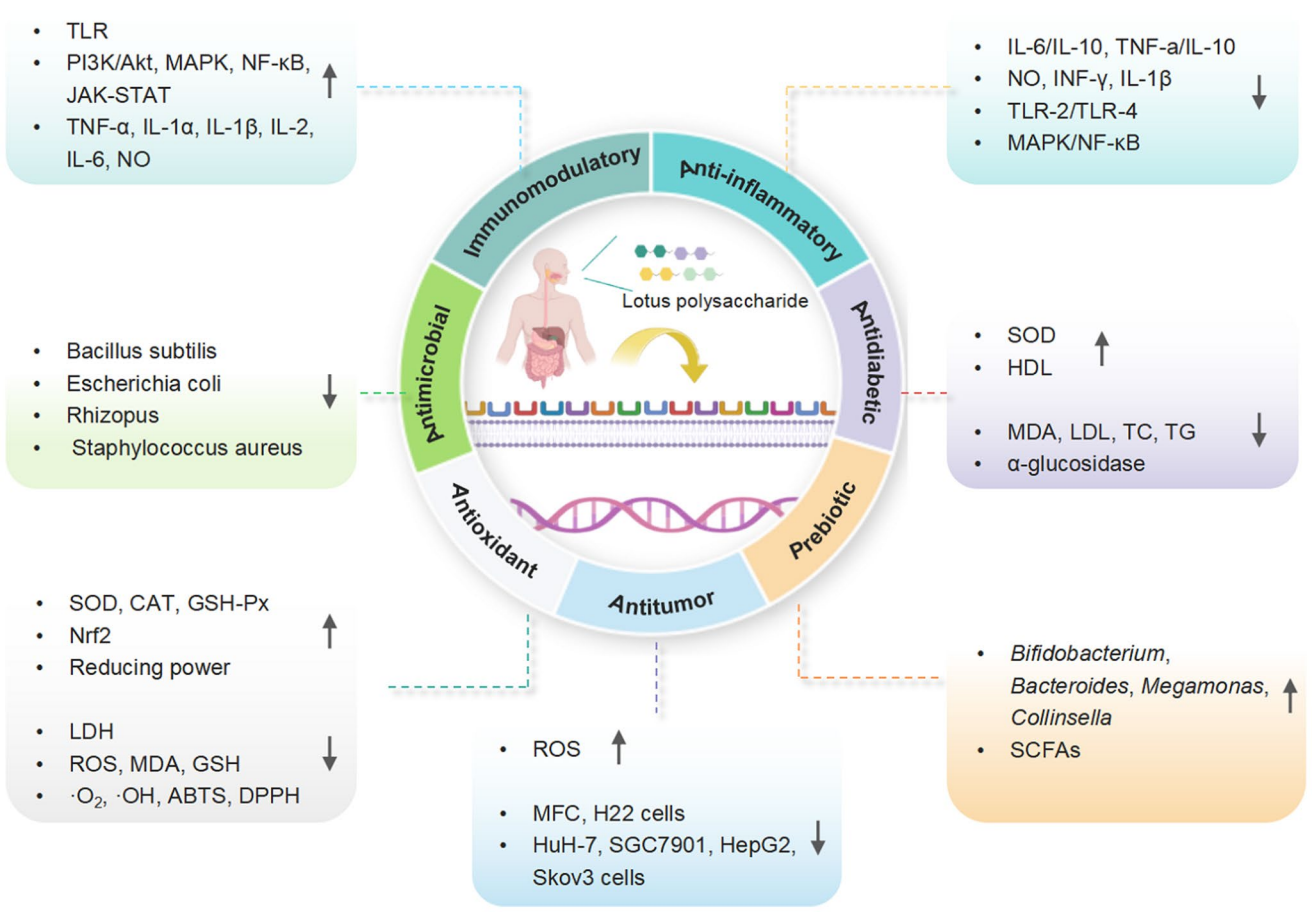
A schematic representation for the biological functions of LPs (created with BioRender.com).

**Figure 5:**
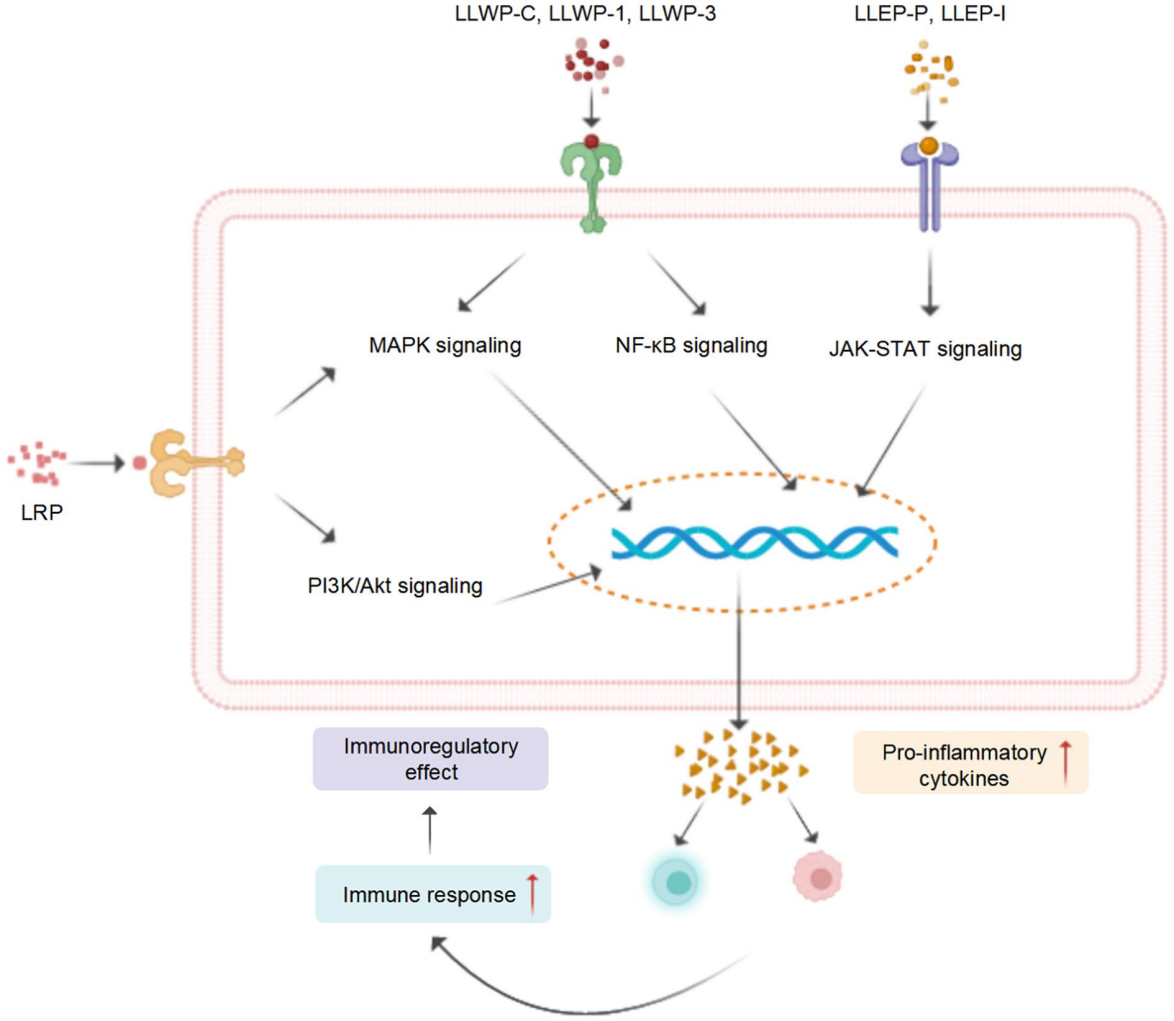
A tentative schematic representation for the proposed mechanisms of immunostimulatory activity of LPs (created with BioRender.com).

**Figure 6:**
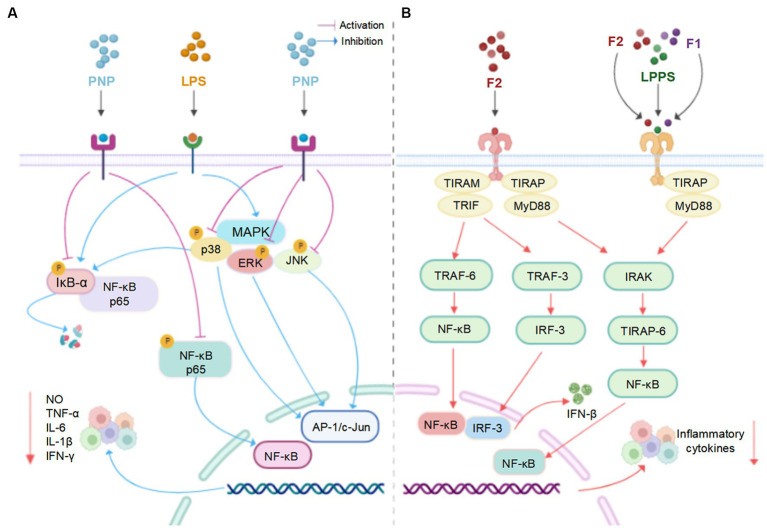
**(A)** The possible mechanism of PNP and LPs inhibiting inflammation in RAW 264.7 cells (66). **(B)** The possible mechanism of LPPS, F1 and F2 inhibiting inflammation in mouse primary splenocytes (94) (created with BioRender.com).

Recent studies revealed that LPs and their derivatives possess multiple bioactivities, including immunomodulatory, antioxidant, anti-inflammatory, antitumor, antidiabetic, and prebiotic activities, which are mediated through interactions with various receptors (7). A comprehensive summary of the bioactivities of LPs is presented in Table 2 and Figure 4, and the mechanisms of immunomodulatory and anti-inflammatory activities are summarized in Figures 5, 6, respectively.

**Table 2:** Summary of biological activities of LPs from the different parts (↓: decrease; ↑: increase).

Biological activities/polysaccharide names	Source	Types	Testing subjects	Doses/duration	Effects/mechanisms	Refs
**Antioxidant activity**						
–	Lotus seed red skin	*In vitro*	·DPPH, ·OH	120 μg/mL, 0.4 mg/mL	·DPPH and ·OH scavenging rates: 41.79, 96.29%, respectively	(82)
LSCP, LSRP	Lotus seed pod	*In vitro*	ABTS^+^,·DPPH, OH	–	Anti-oxidant capability	(10)
–	Lotus seed	*In vitro*	OH	–	·OH scavenging rates: 29.4%	(83)
–	Lotus seed	*In vivo*	Aging model mice induced by D-galactose	0.2 and 0.4 mg/kg/d, for 30 days	SOD, CAT, GSH-PX ↑; LPO ↓	(84)
LSPS, LSPS-1	Lotus seed	*In vitro*	·OH,·O_2_^−^	0.1, 0.2,0.3, 0.4, and 0.5 mg/mL	Anti-oxidant capability	(59)
LSP1, LSP2	Lotus seed	*In vitro*	T-AOC, SOD,·DPPH	2 mg/mL	T-AOC: 51.08, 39.25 U/mL; SOD activity: 30.48, 18.64 U/mL; DPPH scavenging rates: 21.5, 62.99%	(21)
LSP1, LSP2	Lotus seed	*In vivo*	Drosophila model treated with H_2_O_2_	0.5%	Cu Zn SOD, Mn SOD, CAT ↑	(21)
LSSP	Lotus seed	*In vitro*	·DPPH, OH	0.4 mg/mL, 10.0 mg/mL	·DPPH and OH scavenging rates: 59.7, 96.29%, respectively	(50)
–	Lotus plumula	*In vitro*	β-Carotene linoleic acid assay	2–50 mg/mL	Anti-oxidant capability	(56, 85)
–	Lotus plumula	*In vitro*	·DPPH, OH, O_2_^−^, FRAP	–	Anti-oxidant capability	(27)
PNP	Lotus plumula	*In vitro*	RAW264.7 cells	400 μg/mL, for 2 h	ROS, MDA, LDH ↓; SOD, CAT, GSH-Px, GSH ↑	(66)
–	Lotus root	*In vitro*	H_2_O_2_ induced RBC	–	Hemolysis of RBC ↓	(15)
NPh_2_	Lotus root	*In vitro*	RBC, H_2_O_2_ induced RBC	–	Hemolysis of RBC ↓	(86)
–	Lotus root	*In vitro*	·DPPH, OH	100, 200, and 400 μg/mL	Anti-oxidant capability	(87)
LRPs	Lotus root	*In vitro*	·OH, ·DPPH	0.2–1.0 mg/mL, 0.5–2.5 mg/mL	IC_50_ of ·OH and ·DPPH: 0.55, 1.65 mg/mL, respectively	(62)
LRPs, LRPs-complexes	Lotus root	*In vitro*	·DPPH, FRAP	0.05–0.8 mg/mL	Anti-oxidant capability: LRPs > LRPs-phenol complexes	(88)
LRP, CM-LRP	Lotus root	*In vitro*	Fe^2+^, ·OH,·O_2_^−^	1, 2, 4, 8, and 10 mg/mL	Fe^2+^ and ·OH scavenging effect: LRP > CM-LRP; ·O_2_^−^ scavenging effect: CM-LRP > LRP	(89)
LRP1, PLP	Lotus root	*In vitro*	ABTS^+^, ·O_2_, Metal ion	1, 2, 4, 8, and 10 mg/mL	Anti-oxidant capability: PLP > LRP1	(69)
CMLRP	Lotus root	*In vitro*	ABTS^+^, ·O_2_^−^,	1, 2, 4, and 8 mg/mL	ABTS^+^ and ·OH scavenging rates: 52.17 and 85.23%, respectively	(90)
LRP, CM-LRP	Lotus root	*In vitro*	FRAP, ·OH	1, 2, 4, 8, and 10 mg/mL	Anti-oxidant capability: CM-LRP > LRP	(89)
LP30, LP45, LP60, LP75	Lotus root residue	*In vitro*	·DPPH, ·OH	0.8 mg/mL	·DPPH scavenging rates: 42.32, 63.44, 62.88, 80.56%; ·OH scavenging rates: 44.56, 37.91, 32.45, 29.01%	(74)
NNLP-I-I	Lotus leaves	*In vivo*	C57BL/6 mice	10, 20, and 50 mg/kg/d, for 14 days	Regulating the Nrf2-mediated intestinal cellular anti-oxidant defense system	(77)
**Immunomodulatory activity**						
–	Lotus seeds	*In vivo*	CY- induced immunosuppressive mice	400 and 200 mg/kg/d, for 7 days	IL-1α, IL-2, ConA, Splenocytes ↑	(91)
LSSP	Lotus seeds	*In vitro*	RAW264.7 cells	200 μg/mL	NO, TNF-α, IL-6, IL-1β ↑	(50)
LRP	Lotus root	*In vitro*	RAW264.7 cells	12.5, 25, 50, 100, and 200 μg/mL	NO, TNF-α, IL-6, AP-1, NF-κB, ERK, JNK, IκBα, Akt, p85 ↑	(8)
LRP	Lotus root	*In vivo*	Cyclophosphamide induced immunosuppressive mice	20, 40, and 80 mg/kg/d	TNF-α, IL-6 ↑	(8)
LRPs	Lotus root	*In vitro*	RAW264.7 cells	0.05–0.80 mg/mL	TNF-α, NO ↑	(62)
LLWP-1, LLWP-2	Lotus root	*In vitro*	RAW264.7 cells	1, 3, 10, 30, and 100 μg/mL	MAPK/NF-κB pathways, NO ↑	(76)
LRPs, LRPs-phenol complexes	Lotus root	*In vitro*	RAW264.7 cells	200 μg/mL	Macrophage-stimulating effect, NO ↑	(88)
LRP	Lotus root residue	*In vitro*	RAW264.7 cells	12.5, 25, 50, 100, and 200 μg/mL	TNF-α, iNOS, IL-6, JKN, ERK, AKT, IκB phosphorylation, p65, c-Jun, c-fos ↑	(47)
LP30, LP45, LP60, LP70	Lotus root residue	*In vitro*	RAW264.7 cells	50, 100, and 200 μg/mL	Macrophage cell, NO ↑	(74)
LLEP-P, LLEP-P-I	Lotus leaves	*In vitro*	RAW264.7 cells	3, 10, and 30 μg/mL	TLR/JAK–STAT signaling, macrophage immune response genes, cytokines, chemokines, interferon ↑	(16)
WNPP-2-RG	Lotus pollen	*In vitro*	Splenocytes	10, 50, 100, and 200 μg/mL	Lymphocyte, macrophage, NO ↑	(78)
**Anti-inflammatory activity**						
LPPS	Lotus plumula	*In vitro*	Primary splenocytes from NOD mice and BALB/c mice	78, 312, and 1,250 μg/mL	IL-10/IL-6 ↑	(64)
LPPS	Lotus plumula	*In vivo*	NOD mice	0.025, 0.125, and 0.3125%, for 15 weeks	TNF-α/IL-10 and IL-6/IL-10 in the livers ↓	(92)
F1, F2	Lotus plumula	*In vitro*	LPS stimulated RAW264.7 macrophages	0.2, 0.4, 2, 4, 10, and 20 μg/mL	IL-6/IL-10 ↓	(93)
F1, F2	Lotus plumula	*In vitro*	Mouse primary splenocytes LPS	1, 2, 3.9, 7.8, 15.6, 31.3, 62.5, and 125 μg/mL	TLR-2 and/or TLR-4 ↓	(94)
LPWF	Lotus plumula	*In vitro*	LPS-stimulated pri-mary murine microglia	25 μg/mL	IL-1β, IL-6, TNF-α ↓	(9)
PNP	Lotus plumula	*In vitro*	RAW264.7 cells	0–400 μg/mL, for 2 h	NO, TNF-α, INF-γ, IL-1β, IL-6, MAPK/NF-κB pathways ↓	(66)
**Antitumor activity**						
LSPS	Lotus seed	*In vitro*	MFC, HuH-7, and H22 cells	50, 100, and 200 μg/mL, for 48 h	Cell growth ↓	(46)
LSPS	Lotus seed	*In vivo*	H22 Mice Hepatocellular Carcinoma Model	50, 100, and 200 mg/kg/d, for 14 days	Cell growth ↓, the highest inhibition rate of 45.36% (200 mg/kg)	(46)
-	Lotus root	*In vitro*	SGC7901 and HepG2 cells	100, 200, and 400 μg/mL	Cell growth ↓	(87)
LRPs	Lotus root	*In vitro*	HepG2 and SGC7901 cells	0.80 mg/mL	Cell growth ↓, the inhibition rates were 44.25 and 36.30%, respectively	(62)
PLP	Lotus root	*In vitro*	Skov3 cells	100, 200, 300, 400, and 500 μg/mL, for 24 h	Cell growth, SOD ↓	(69)
**Antidiabetic activity**						
LPPS	Lotus plumula	*In vivo*	NOD mice	0.025, 0.125, and 0.3125%, for 15 weeks	HDL-C, LDL-C, TC ↓	(95)
NNP-2	Lotus plumula	*In vitro*	Insulin-resistant HepG2 cells	0.1–3 mg/mL, for 24 h	IRS1/PI3K/Akt pathway ↑	(96)
–	Lotus root	*In vivo*	Alloxan-induced diabetic rats	50, 100 and 200 mg/kg, for 28 days	Blood glucose level, SOD ↓; glucose tolerance, MDA ↑	(97)
LLP	Lotus leaf	*In vivo*	Rats with gestational diabetes mellitus	50 and 100 mg/kg, for 14 days	FBG, FINS, TC, TG, LDL-C, SOD ↓; HDL-C, GPx, GSH, GDM ↑	(98)
LLP-M	Lotus leaf	*In vitro*	α-glucosidase	2.0–6.0 μg/mL	IC_50_ of α-glucosidase: 1.90 ± 0.02 μg/mL	(99)
**Prebiotic activity**						
LOS3-1, LOS4	Lotus seed	*In vitro*	*Lactobacillus acidophilus*	–	*Lactobacillus acidophilus* ↑	(65)
NNP-2	Lotus plumula	*In vitro*	*Lactobacillus* and *Bifidobacterium*	0.5, 1, and 2% w/v, for 2 days	*Lactobacillus*, *Bifidobacterium* ↑	(96)
LLPI	Lotus leaf	*In vitro*	–	–	*Bacteroides*, *Bifidobacterium*, *Megamonas*, *Collinsella* acetic, propionic, and butyric acids↑	(100)
LRP	Lotus root	*In vitro*	–	–	Firmicutes/Bacteroidetes, PH ↓; *Bifidobacterium* ↑	(61)
**Antimicrobial activity**						
–	Lotus leaf	*In vitro*	*Bacillus subtilis*, *Escherichia coli*, *Staphylococcus aureus*, *Proteus species*, *Rhizopus* sp.*, Aspergillus sp., Penicillium sp., Mucor sp., Saccharomyces cerevisiae*	2.5, 5, 10, 20, 40 and 80 mg/mL, for 1 mL	MIC of *Staphylococcus aureus* - 20 mg/mL, *Escherichia coli and Proteus species*, and *Rhizopus* sp - 40 mg/mL, *Bacillus subtilis* and *Aspergillus sp*-80 mg/mL	(101)
–	Lotus leaf	*In vitro*	*Escherichia coli*, *Bacillus subtilis*, *Aspergillus niger*, *Saccharomyces cerevisiae*	1.25, 2.5, 5, 10, 20, and 40 mg/mL	MIC of *Escherichia coli-*10 mg/mL, *Bacillus subtilis* - 20 mg/mL, *Aspergillus niger-*40 mg/mL	(102)
–	Red skin of lotus seed	*In vitro*	*Escherichia coli*, *Staphylococcus aureus*, *Aspergillus niger, Aspergillus flavus*	10, 20, 30, 40, and 50 mg/mL	MIC of *Escherichia coli*, and *Staphylococcus aureus* - 20 mg/mL	(82)
**Inhibition of pancreatic lipase activity**						
–	Red skin of lotus seed	*In vitro*	Pancrelipases	0.15 g/mL	Inhibition rate: 94.61%	(81)
**Antiosteoporotic**						
LIEP	Lotus leaf	*In vivo*	Ovariectomized mice	30 and 100 mg/kg, for 28 days	C-Fos/NFATc1 ↓	(103)
**AntiHIV**						
LB2	Lotus leaf	*In vitro*	Recombinant HIV-1 PR of *Escherichia coli* origin	–	HIV-1 RT, HIV-1 3′-processing, TNFα ↓	(71, 72)

### 5.1. Antioxidant activity

As representative plant polysaccharides, LPs exhibited excellent antioxidant activities in β-carotene linoleic acid, superoxide anion (·O2-), hydroxyl (·OH), 2, 2-azinobis-6-s-(3-ethyl benzothiazoline sulfonic acid) (ABTS), and 1,1-dipheny1-2-picrylhydrazyl (·DPPH) assays (10, 50, 59, 62). LPs also exhibited significant reducing power and anti-lipid peroxidation activity (27, 56, 69). Moreover, the root LPs effectively prevented the oxidative damage of red blood cells induced by H_2_O_2_ (15), which is consistent with Yan et al. (86). A plumula LPs (PNP) effectively prevented oxidative damage in RAW264.7 cells by reducing the production of lactate dehydrogenase (LDH), reactive oxygen species (ROS), and malondialdehyde (MDA), improving the activity of glutathione peroxidase (GSH-Px), superoxide dismutase (SOD), and catalase (CAT), and stimulating the production of GSH (66).

The antioxidant activity of LPs has also been investigated *in vivo*. In a study conducted on D-galactose-induced aging mice, oral administration of seed LPs (PLPs) (0.2 and 0.4 g/kg/d) for 30 days resulted in a significant increase in the activity of SOD, CAT, and GSH-Px in blood, Concurrently, it led to a decrease in the levels of lipid peroxide (LPO) in the plasma, liver, and heart (84). Seed LPs extended the lifespan of *Drosophila melanogaster* via upregulating the expression level of CuZnSOD, CAT, and MnSOD genes (21). Additionally, leaf LPs (NNLP-I-I) exhibited excellent antioxidant properties by regulating the nuclear factor erythroid 2-related factor (Nrf2) and improving the intestinal cellular antioxidant defense system (77). Although multiple studies have provided evidence of the antioxidant actions of LPs *in vitro*, there is a scarcity of *in vivo* investigations. Thus, it is imperative to conduct further comprehensive research to more thoroughly understand the antioxidant mechanism of LPs.

### 5.2. Immunomodulatory activity

The immune response is recognized as the primary defense mechanism against cancer and infections (104). For instance, the seed LPs (LSSP) exhibit significant immunomodulatory activity by stimulating the production of nitrous oxide (NO), interleukin-1β (IL-1β), IL-6, and tumor necrosis factor-α (TNF-α), in activated RAW 264.7 cells (50). Four root PLs (LP30, LP45, LP30, and LP60) induced the release of NO and enhanced the activity of macrophages (74). Song et al. (76) investigated the mechanism of macrophage activation of crude leaf LPs (LLWP-C) and two derived ingredients (LLWP-1 and LLWP-3), which effectively improved the phagocytic and secretory effects of macrophages via upregulating the mitogen-activated protein kinase (MAPK) and nuclear factor κB (NF-κB) pathways. Another leaf LPs (LLEP-P-I) launched the immune responses of macrophages via upregulating the Janus tyrosine kinase/toll-like receptor (JAK–STAT) and toll-like receptor (TLR) (16).

In the cyclophosphamide (CY)-induced immunosuppressed mice, the seed LPs enhanced the immunocompetence by promoting the secretion of IL-1α and IL-2 and reducing the production of soluble interleukin-2 receptor (SIL-2R) (91). A root LPs (LRP) promoted the production of IL-2 and TNF-α in CY-induced immunosuppressive mice, possibly by activating the MAPK and phosphoinositide 3-kinase/protein kinase (PI3K/Akt) signaling pathways (8), which agrees with Sun et al. (47). These studies indicated that LPs possess potent immunomodulatory activity and could be developed as potential dietary supplements or immunomodulators.

### 5.3. Anti-inflammatory activity

Accumulated studies reveal that LPs exhibited anti-inflammation effects by regulating pro−/anti-inflammatory cytokine levels. In a study conducted on non-obese diabetics (NOD) mice, the oral administration of plumula LPs (LPPS) for 15 weeks markedly decreased the secretion ratios of IL-6/IL-10 cytokines in splenocytes, and dose-dependently decreased absolute weights of the enlarged spleens (64). Furthermore, LPPS exhibited a marked downregulation of IL-6/IL-10 and TNF-a/IL-10 expression levels in the livers of NOD mice (92).

*In vitro*, the anti-inflammatory activities of two plumula LPs (F1 and F2) were assessed in splenocytes and lipopolysaccharide (LPS)-stimulated RAW264.7 cells. The secretion ratios of IL-10/IL-6 cytokine were remarkably increased after the treatment of F1 and F2, especially the latter, in a dose-dependent manner. The mechanisms of action could be decreasing the production of TLR-4 or TLR-2 (93, 94). Moreover, plumula LPs (PNP) effectively decreased the secretion of IL-1β, IL-6, INF-γ, TNF-α, and NO in RAW264.7 cells, possibly through the inhibition of the MAPK/NF-κB signaling pathways (66). In addition, a specific type of plumula LPs (LPWF) significantly inhibited the production of TNF-α, and IL-6, IL-1β in the primary murine microglia stimulated by LPS (9).

In brief, LPs demonstrate considerable anti-inflammatory effects both *in vivo* and *in vitro*, suggesting their potential as therapeutic agents for inflammation. However, existing literature on the mechanism and SARs underlying the anti-inflammatory activity of LPs is limited. Thereby, it is necessary to further examine these SARs and their specific reaction mechanism.

### 5.4. Antidiabetic activity

Diabetes mellitus (DM) is a prevalent metabolic disease induced by an abnormal increase in blood glucose levels, which has become the third leading cause of mortality worldwide (104). LPs exhibited great potential in treating DM. Luo et al. (97) studied the hypoglycemic activity of root LPs using an alloxan-induced diabetic mice model. The oral administration of root LPs for 28 days reduced the level of glucose lipid, improved glucose tolerance, and increased the body weight of diabetic mice. Additionally, root LPs enhanced the SOD activity and decreased MDA levels in the kidney, liver, and pancreas of diabetic mice. In NOD female mice, after 15 weeks of administration of plumula LPs (LPPS), the number of pancreatic islet cells increased, the level of low-density lipoprotein cholesterol (LDL-C) and total cholesterol (TC) significantly decreased, and the level of high-density lipoprotein cholesterol (HDL-C) improved (95). Furthermore, Zeng et al. (98) studied the hypoglycemic effect of the leaf LPs (LLP) in pregnant rats with gestational DM (GDM). Oral administration of LLP effectively improved the lipid profile of GDM rats, as evidenced by significantly decreased serum levels of LDL-C, TC, and triglyceride, except for HDL-C.

Le et al. (96) isolated a plumula LPs (NNP-2), which inhibited α-glucosidase with IC_50_ 97.32 μg/mL. Protein expression analysis and real-time PCR showed that NNP-2 could ameliorate insulin resistance in HepG2 cells by regulating the IRS1/PI3K/Akt pathway. Four fractions of leaf LPs (LLP-RF, LLP-V, LLP-M, and LLP-H) demonstrated remarkable antiglycation and α-glucosidase inhibitory activity *in vitro* (99). These findings suggest that LPs possess potent hypoglycemic activity and are potential dietary supplements or hypoglycemic agents.

### 5.5. Antitumor activity

LPs have demonstrated remarkable inhibitory effects on cell proliferation in various cancers, e.g., ovarian carcinoma, gastric and liver cancer, *in vivo* and *in vitro*. The administration of seed LPs (LSPS) effectively inhibited the growth of mouse hepatocarcinoma H22 cells, human liver cancer HuH-7 cells, and mouse gastric cancer MFC cells. Moreover, *in vivo* experiments showed that LSPS significantly suppressed tumor cell proliferation in H22-bearing mice with inhibition rates of 17.9.%, 39.60, and 45.36%, respectively (50, 100, and 200 mg/kg/d, 14 days) (46). In another study, Yi et al. (87) isolated 39 LPs from peels, fleshes, and nodes of 13 lotus roots and found that all LPs can notably inhibit the growth of HepG2 and SGC7901 cells. Furthermore, a specific type of LPs derived from the root (LRP) significantly suppressed the proliferation of HepG2 and SGC7901 cells in a dose-dependent manner at 0.05–0.80 mg/mL (62). Yan et al. (69) evaluated the anticancer activity of phosphorylated root LPs (LRPs) in human ovarian cancer Skov3 cells, and found that LRPs effectively inhibited Skov3 proliferation and induced reactive ROS production. Despite significant advances in understanding the antitumor activity of LPs, the underlying mechanism remains limited. As a result, further research is needed to comprehensively assess their potential therapeutic applications.

### 5.6. Prebiotic activity

LPs have garnered interest as potential prebiotics due to their ability to modulate the intestinal flora by facilitating the fermentation and growth of beneficial bacteria, such as *Bacteroides*, *Bifidobacterium*, *Megamonas*, and *Collinsella*, consequently enhancing the production of short-chain fatty acids (SCFAs) (105). Studies have shown that plumula LPs (NNP-2) improved the relative abundance of probiotics, specifically *Lactobacillus acidophilus* and *Bifidobacterium adolescentis* (96). Additionally, two lotus seed oligosaccharides (LOS3-1 and LOS4) effectively increased the survival rate of *L. acidophilus* (65). Recently, Guan et al. (60) explored the prebiotic effect of the root LPs (LRP) utilizing an *in vitro* fermentation model. LRP demonstrated the capacity to modulate intestinal microbiota by promoting *Bifidobacterium*’s growth and altering the ratio of *Firmicutes*/*Bacteroidetes*, thereby promoting the generation of SCFAs (butyric, propionic, and acetic) (61). Besides, leaf LPs (LLPI) selectively improved the abundance of beneficial microbes, including *Megamonas, Bacteroides*, *Bifidobacterium*, and *Collinsella*, and effectively promoted the production of SCFAs (100). These findings underscore the potential of LPs to improve the intestinal micro-environment and highlight their prebiotic properties. Further research is required to assess the digestive and glycolytic characteristics of LRPs in future studies.

### 5.7. Antimicrobial activity

Leaf LPs had an antimicrobial effect against *Escherichia coli* and *Rhizopus* with minimal inhibitory concentration (MIC) of 20 and 40 mg/mL, respectively (101). An *in vitro* antimicrobial screening assay revealed the growth inhibitory efficiency of the seed LPs (20 mg/mL) on the growth of *Staphylococcus aureus* and *E. coli* (82). Moreover, the leaf LPs effectively reduced the growth of *E. coli* and *Bacillus subtilis*, although they had little inhibition on *Yeast* and *Aspergillus niger* (102). However, there are a limited amount of studies on the antimicrobial activity of PLPs *in vivo*, and more studies are warranted.

### 5.8. Other bioactivities

LPs have demonstrated various additional biological activities. Specifically, three types of root LPs (LB2, L2f-2, and L2f-3) exhibited significant inhibition of HIV-1 reverse transcriptase and integrase L2f-3. Notably, LB2 directly inhibited HIV-1 via reducing the expression of TNF-α (71, 72). At 0.15 g/mL, the inhibition rate of the seed LPs on pancreatic lipase reached 94.61%, and its inhibition constant was 0.0736 g/mL (81). Furthermore, leaf LPs (LLEP) demonstrated antiosteoporotic effects in ovariectomized mice. Oral administration of LLEP at doses of 30 and 50 mg/kg/d for 4 weeks remarkably ameliorated the estrogen deficiency-induced bone loss, potentially through the down-regulation of c-Fos/NFATc1 expression (103).

## 6. Structure-activity relationships

The activity of plant polysaccharides is strongly associated with their structural feature (45). Some studies showed that the monosaccharide, *Mw*, composition, glycosidic linkage, chemical conformation, and structural modification are critical influencing factors for the bioactivities of LPs (38).

### 6.1. Monosaccharide composition

Several studies have demonstrated that the monosaccharide composition of LPs is closely related to their biological activity, particularly in terms of Gal, Ara, and Man (62, 78, 87). For instance, Yi et al. found that the immunostimulatory activity of root LPs (LRPs) is associated with the presence of Gal and Man side chains, while the antitumor activity of LRPs may be related to the branched Man residues (62). Moreover, Ara content exhibited a positive correlation with the ferric-reducing antioxidant power (FRAP) and DPPH radical scavenging activity of LRPs, and the Gal content had a positive effect on the ·OH scavenging effect (87). Furthermore, Li et al. reported that hydrolyzed LPs (RG-8H-P) exhibited increased phagocytic activity within macrophages compared to the original LPs. This can be attributed to alterations in the spatial structure and conformation of Gal side chain residues subsequent to the hydrolysis of the original polysaccharides (78). These alterations likely enhance the binding affinity between RG-8H-P and its receptors, thereby facilitating the phagocytosis, digestion, and metabolism of foreign materials by macrophages (106). Similar observations were documented for the polysaccharides derived from *Solieria chordalis* (107). These findings indicate the significance of monosaccharide composition in understanding the biological activity of LPs.

### 6.2. Molecular weight

Numerous studies have demonstrated a correlation between the *Mw* of LPs and their biological activity (60). Specifically, it has been observed that low-*Mw* F2 (25.7 kDa) displays more potent anti-inflammatory effects than F1 (> 2000 kDa) (94), presumably owing to the improved permeability of low-*Mw* LPs, facilitating their penetration through the cell membrane (23). Additionally, LPs derived from leaves with low-*Mw* exhibit a more pronounced activation effect on RAW264.7 macrophages (76) as well as superior antioxidant activity, α-amylase inhibition, and α-glucosidase inhibition (12), aligning with previous findings regarding the polysaccharides of *Polygonatum* (108). Notably, LPs with low-*Mw* (LOS3-1 and LOS4) display significant prebiotic activity (65), likely due to their enhanced absorption and utilization by *L. acidophilus* (109). However, the exact mechanism underlying this discrepancy remains elusive, warranting further studies.

### 6.3. Structural modifications

Appropriate chemical modifications have been shown to enhance the efficacy of LPs and broaden their potential applications (39). Recent studies showed that phosphorylation and carboxymethylation could significantly improve the scavenging ability of LRPs on ABTS, ·OH radicals, and ferrous ions (69, 89). This enhancement is likely attributed to the introduction of carboxymethyl and phosphate groups, which alter the water solubility and spatial conformation of LRPs, thereby influencing their biological activity (110). Furthermore, Yi et al. (88) observed a significant enhancement in the FRAP capacity and ·DPPH radical scavenging effect of phenolic compound-modified LRPs. The extent of enhancement was positively correlated with the binding ratio of phenolic compounds, possibly due to the presence of multiple hydroxyl groups with unpaired electrons in these compounds (110). Yuan et al. (111) found that the binding of Fe (III) with LRPs can significantly enhance the antioxidant activities of LRPs. It can also alleviate the symptoms of iron deficiency anemia in mice by impacting the gut microbiome and regulating the biosynthesis of steroid hormones.

Due to the scarcity of literature on the chemical structures and structural modifications of LPs, it is challenging to explain the SARs of LPs. Undoubtedly, comprehensively understanding these aspects would propel the development of LPs-based dietary supplements and therapeutic medications. Therefore, it is urgently necessary to expand current scientific research in this field.

## 7. Applications

In recent years, LPs and their derivatives have not only been used to treat and prevent disease but also used to develop nutritional enhancers and dietary supplements. LPs have gained widespread utilization as natural active ingredients in health products. For instance, a composite functional beverage was prepared using roots LPs, vitamin C, sucrose, oligosaccharide maltose, citric acid, NaCl, food color, and essence, which has a significant antifatigue effect (112). Due to the ability to promote the growth of beneficial bacteria and stimulate the production of SCFAs (61, 65, 100), LPs hold promising potential as prebiotics in the food industry. Additionally, LPs exhibit significant immunomodulatory activity (16, 74), making them a favorable food supplement for individuals with compromised immune function. Recent studies showed that lotus pectic polysaccharides possess characteristics that contribute to satiety and hinder digestion, rendering them suitable for the development of weight loss products (13).

In addition, LPs can also be used as additives in cosmetics because of their anti-oxidant and anti-senescence activities (21, 91). Besides, LPs can be applied in the research and development of new drugs because of their biological activity, such as anti-inflammatory, antidiabetic, and antiosteoporotic activities. LRPs could be used as a potential drug carrier to develop new systems for drug release, particularly colon-delivery systems (113).

## 8. Safety assessment

It is critical to evaluate the cytotoxicity and adverse effects before developing any product for food and medical treatments. To date, various beneficial effects of LPs have been extensively studied. However, studies investigating the safety and toxicity of LPs are lacking. In H22 tumor-bearing mice, oral administration of seed LPs (LSPS) (50, 100, and 200 mg/kg) for 14 days did not alter the blood parameters, e.g., platelets, hemoglobin, and red blood cells (46). Zeng et al. (98) studied the toxicity of leaf LPs (LLP) in rats and found that the neurologic and behavioral alterations of LLP-treated rats were regular, and no death or harmful effect was observed during the experiment. *In vitro* studies have shown that the plumula LPs (PNP) of 25–400 μg/mL had no toxicity on RAW264.7 cells (66). While these findings suggest the potential safety of LPs, it is essential to note that their toxicity studies remain underdeveloped. Consequently, additional research involving clinical trials and animal experiments is needed to further verify their safety and toxicity.

## 9. Conclusion and future perspective

Numerous studies have focused on the extraction, separation, purification, structural identification, and pharmacological effects of LPs. The most common extraction method for LPs is HWE, while UAE and MAE improve the extraction rate of LPs. LPs are classified as heteropolysaccharides, comprising monosaccharide units such as Glc, Gal, Ara, Xyl, Man, and Rha. These polysaccharides possess diverse bioactivities, including immunoregulatory, anti-inflammatory, antioxidant, antidiabetic, antitumor, prebiotic, and antimicrobial, and can be used as promising functional food supplements or therapeutic agents. It is worth noting that the biological activities of LPs are influenced by their structural characteristics, especially the *Mw*, monosaccharide composition, and glycosidic bonds, among other factors.

Despite the opulent results of LPs, there are still some critical issues that need to be addressed. Firstly, new methods have been used for the extraction of LPs. However, industrial production needs more simple, efficient, and less expensive methods to produce high-quality LPs. Different extraction methods should be combined to develop methods suitable for industrial production. Secondly, the current focus of structural analyses of LPs predominantly revolves around their primary structure, such as *Mw*, monosaccharide composition, glycosidic bonds, etc. The advanced structure (spatial conformation) of LPs must be elucidated by circular dichroism, scanning tunneling microscopy, and X-ray diffraction. Thirdly, exploring SARs of LPs is still in its preliminary stages. The relationships between the structure (including *Mw*, monosaccharide composition, type of glycosidic linkages, and chemical conformation) and the activity of LPs remain unclear and require further exploration. Fourthly, it is worth noting that there are remarkable differences between the different sources and medicinal parts of lotus in the biological activity, structural features, and content of LPs. It is necessary to systematically investigate the different parts and kinds of LPs, which is critical for quality control. Fifthly, the action mechanism, dosage, usage, course, and safety of LPs are poorly understood, and more pharmacological experiments and acute/chronic toxicity studies should be conducted to explore the underlying mechanisms, optimal dosage, reliability, and effectiveness. Lastly, exploring specific structural modifications, such as hydroxylation, selenization, sulfation, and olefination, should be pursued to enhance the bioactivity of LPs, which is one of the critical directions for future research.

## Author contributions

GD, JW, and JZ: writing – original draft. CX, YW, and BD: conceptualization, supervision, project administration. All authors contributed to the article and approved the submitted version.

## Funding

This work was supported by the Yunnan academician expert workstation [202105AF150053, 202205AF150026], the key technology projects in the Yunnan Province of China [202002AA100007], and the Yunnan Xingdian talent support plan [YNWR-QNBJ-2020251].

## Conflict of interest

The authors declare that the research was conducted in the absence of any commercial or financial relationships that could be construed as a potential conflict of interest.

## Publisher’s note

All claims expressed in this article are solely those of the authors and do not necessarily represent those of their affiliated organizations, or those of the publisher, the editors and the reviewers. Any product that may be evaluated in this article, or claim that may be made by its manufacturer, is not guaranteed or endorsed by the publisher.
